# Correspondence

**Published:** 1859-11

**Authors:** 


PHILADELPHIA, Oct 10, 1859.

MESSRS. EDITORS:—Since my last communication to you, I have, after attending the Convention at Niagara, spent several weeks in the country upon a farm, in the pursuit of health, and while there, was painfully impressed with the ignorance and consequent neglect on the part of the farmers in regard to their teeth. I was astonished to find that more than half of all I met, including many of both sexes, just reaching their majority, had badly decayed teeth, or what was worse, no teeth visible at all, presenting an unsightly, and in some cases, a disgusting appearance.

Now, how shall we account for the early decay of the teeth of a class of the community who live naturally, enjoying full bodily health, with good appetites and good digestion, with plenty of out-door exercise to develop and strengthen the entire system? Of one thing, however, I am quite sure, which is, that habits of *cleanliness of the teeth* would do much toward preserving them, and likewise render their owners more companionable; a tooth-brush is rarely heard of among them. But how to reach them, to impress upon their attention this great necessity of *cleanliness*, is the question, and but one way suggests itself to me, which is this, that every village dentist communicate to the local newspaper, short but plain and attractive essays (free from technicalities) upon the importance of preserving the teeth, their use in the animal economy, with some general directions as to the use of the brush, etc. This should all be free from anything of an advertising character, and thus have more weight, and the more likely secure publication in the newspaper, as a matter of general interest.

Now, all this done from pure motives of good to the public, uninfluenced by personal interest or aggrandizement, *must* prove beneficial to those by whom the writer is surrounded, and he be amply repaid eventually, as surely as the farmer reaps where he sows.

This subject might be elaborated, but I think enough has been said to make it perfectly intelligible, and to show that by the plan proposed, the teeth of the young of the present generation throughout the agricultural districts, and especially in the newly settled countries, might be greatly improved and many preserved; the dentist make himself more useful, both as practitioner and writer, and ultimately our journals be better sustained by contributions (for it will be observed the plan results in the education of practitioners as writers), and thus the profession of dentistry accomplish more fully its important mission, by extending the field of its usefulness, and by eventually bringing all under its wholesome influence.

Surely such a result is desirable, and I as firmly believe may be brought about, to a great extent at least, by the means proposed. If it should strike you favorably, give it the sanction and weight of your editorial influence.

If not considered heretical, I would suggest that the last meeting of the American Dental Convention was not quite *up* in interest to any one of the four previous meetings. This I think, will be noticed by a perusal of the reported discussions, and more fully, by comparison with those of previous meetings.

This evidence of declining interest (for such I take it to be) gives additional promise of success to the new organization, "The American Dental Association," in plainly indicating the necessity for a more thorough and substantial organization—one having more ties and attractions, and giving promise of more positive and permanent usefulness than can be hoped for from one of the—I may say—ephemeral character of the American Dental Convention.

I observe New York has given birth to another dental organization. This must be somewhere about the fourth or fifth child of the same parentage, none of which, however, long survived their birth. May this last be spared to cheer its so often and so sadly bereaved progenitors, and prove a blessing to them and their posterity.

I notice with pleasure the absence, to a great extent, of exhibitions of dentistry from our Pennsylvania State Agricultural Fair.

There were but three exhibitors in this department, one of porcelain teeth of very ordinary quality, one of mechanical dentistry, gotten up for show, and one of the porcelain plate process. The Committees on "Stallions," or imported stock, are hardly the men to pass judgment on dental matters. Exhibitions of dentistry at such places should be frowned down as humiliating, as well as detrimental to the profession.

Of the movements of some of the profession, I notice the return of Dr. Theodore S. Evans to this country, after an absence of some eight years. His visit here will be brief, and is one, I suspeet, of a very tender nature. I note also the departure of Dr. W. M. Wright, of Pittsburg, for London, where he designs practicing his profession for a short time, at the solicitation of an influential friend and patron, a resident of London.

Such events are highly complimentary to American dentistry, and in this instance is peculiarly appropriate and well merited. Yours, O. U. C.

